# Implementing a survey for patients to provide safety experience feedback following a care transition: a feasibility study

**DOI:** 10.1186/s12913-019-4447-9

**Published:** 2019-08-30

**Authors:** Jason Scott, Emily Heavey, Justin Waring, Aoife De Brún, Pamela Dawson

**Affiliations:** 1 Faculty of Health and Life Sciences, Northumbria University, Newcastle upon Tyne, UK; 2 Department of Behavioural and Social Sciences, University of Huddersfield, Huddersfield, UK; 3 Health Services Management Centre, University of Birmingham, Birmingham, UK; 4 School of Nursing, Midwifery and Health Systems, University College Dublin, Dublin, Ireland; 5 PD Education and Health Consulting Ltd, Newcastle upon Tyne, UK

**Keywords:** Patient safety, Care transitions, Feasibility, Patient experience

## Abstract

**Background:**

The aim was to determine the feasibility of implementing a patient safety survey which measures patients’ experiences of their own safety relating to a care transition. This included *limited-efficacy testing,* determining *acceptability* (to patients and staff), and investigating *integration* with existing systems and practices from the staff perspective.

**Methods:**

Mixed methods study in 16 wards across four hospitals, from two English NHS Trusts and four clinical areas; cardiology, care of older people, orthopaedics, stroke. Limited-efficacy testing of a previously validated survey was conducted through collection of patient reports of safety experiences, and thematic comparison with staff safety incident reports. Patient acceptability was determined through analysis of survey response rates and semi-structured interviews. Staff acceptability and integration were investigated through analysis of survey distribution rates, semi-structured interviews and focus groups.

**Results:**

Patients returned 366 valid surveys (16.4% response rate) from 2824 distributed surveys (25.1% distribution rate). Older age was a contributing factor to lower responses. Delays were the largest safety concern for patients. Staff incident report themes included five not present in the safety survey data (*documentation, pressure ulcers, devices or equipment, staffing shortages, and patient actions*). Patient interviews (*n* = 28) identified that providing feedback was acceptable, subject to certain conditions being met; *cognitive-cultural* (patient understanding and prioritisation of safety), *structural-procedural* (opportunities, means and ease of providing feedback without fear of reprisals), and *learning and change* (closure of the feedback loop). Staff (*n* = 21) valued patient feedback but barriers to collecting and using the feedback included resource limitations, staff turnover and reluctance to over-burden patients.

**Conclusions:**

Patients can provide meaningful feedback on their experiences and perceptions of safety in the context of care transitions. Providing this feedback was acceptable to some patients, subject to certain conditions being met. Safety experience feedback from patients was also acceptable to staff; quantitative data was perceived as useful to identify potential risks, and qualitative data informed types of changes required to improve care. However, patient feedback was not integrated into any quality improvement initiatives, suggesting there are still significant challenges to healthcare teams or organisations utilising patient feedback, particularly in relation to care transitions.

**Electronic supplementary material:**

The online version of this article (10.1186/s12913-019-4447-9) contains supplementary material, which is available to authorized users.

## Background

Patient transitions across organisational boundaries are high in risk [1–4] and haphazard [5], often as the result of inconsistent care coordination between healthcare organisations or teams [6], and lack of patient involvement in the planning process [7]. This is particularly problematic when different health and social care organisations, and their accompanying structures and processes, are required to work together in order to provide integrated, patient-centred, high-quality care [8]. In England, healthcare policy is placing an increasing emphasis on greater integration between health and social care services [9, 10]. However, there are many challenges associated with delivering safe, integrated care, including a lack of alignment between health and social care organisations in their understanding of, and approaches to, safety [11]. Furthermore, providing safe care during discharge from hospital, which is just one stage of a patient’s transition, is rarely perceived by clinicians to be a linear or causal occurrence. Safety is instead the result of communication and collaboration within a complex system of multiple organisations and boundaries [12], which can also include the patient themselves.

The patient is often the only point of continuity across the care pathway and therefore has a unique perspective of the transition that is not otherwise available to clinicians or staff [7, 13, 14]. When willing and able [15], patients are believed to have a role in improving their own safety during transitions, which includes the identification and reporting of their own safety [16] and increased involvement in the handover process itself [17]. Patients should be involved at all levels in their own safety [18], with this involvement falling into three categories: informing a management plan, monitoring and ensuring safe delivery of treatment, and making systems safer [19], the latter of which includes reporting on experiences of safety. Efforts are now being made to implement or test the implementation of various systems to obtain patient reports of safety incidents [20, 21]. However the efficacy of such systems is limited, particularly due to the challenges of making these systems routine for patients to complete, which can require considerable staff input [22, 23], and limited evidence of successfully using patient feedback for organisational learning [24, 25]. An alternative approach has been to link data sets at the patient level from across the patient’s journey, which provides a more holistic picture of safety than analyses of individual events [26], but this still does not fully take into account the patient’s experience.

By involving patients in their own safety, healthcare professionals can encourage them to act as an extra safeguard within the healthcare system [16, 27], which is in line with the systems approach to safety [28]. However in doing so, it is important to acknowledge that the definitions of safety differ between the patient and healthcare professional [29–31], .and it is only the patient who can identify and report on feeling safe or unsafe in relation to their own definition of safety. There is also an important distinction to make between reporting safety incidents and providing feedback on experiences of safety. The former is based on medically-defined events that have led or had the potential to lead to harm to the patient, whilst the latter is based on the patients’ own feelings of how safe they felt, regardless of the risk of harm. There is a strong link between patient experience, safety and clinical effectiveness [32], and it is proposed that patient feedback on safety experiences can provide a source of data that highlights latent conditions within care transitions. As such, there is a need to explore how patients can be enabled and supported to provide feedback on their safety experiences relating to their care transition.

## Methods

### Aims and objectives

The aim of this study was to determine the feasibility of implementing a patient safety survey which measures patients’ experiences of their own safety relating to care transition, and in particular the discharge, journey and arrival stages of a transfer out of hospital. Three ‘areas of focus’ that have been identified to be important to feasibility studies [33] were explored: *limited-efficacy testing, integration,* and *acceptability* (to patients and staff). Specific research objectives included:

1. Test the limited-efficacy of the survey by measuring experiences of safety relating to a care transfer following discharge from hospital, including a comparison of how these experiences relate to staff safety incident reports.
2. Determine acceptability of the survey to patients using response rates as an indicator, and reflecting on semi-structured interviews with patients that were previously published [34]
3. Investigate the integration of the survey with existing systems and practices, and acceptability of the survey amongst healthcare teams to the reporting tools and reports of safety, and the limited-efficacy of using feedback for organisational learning.

### Study design

**Fig. 1:**
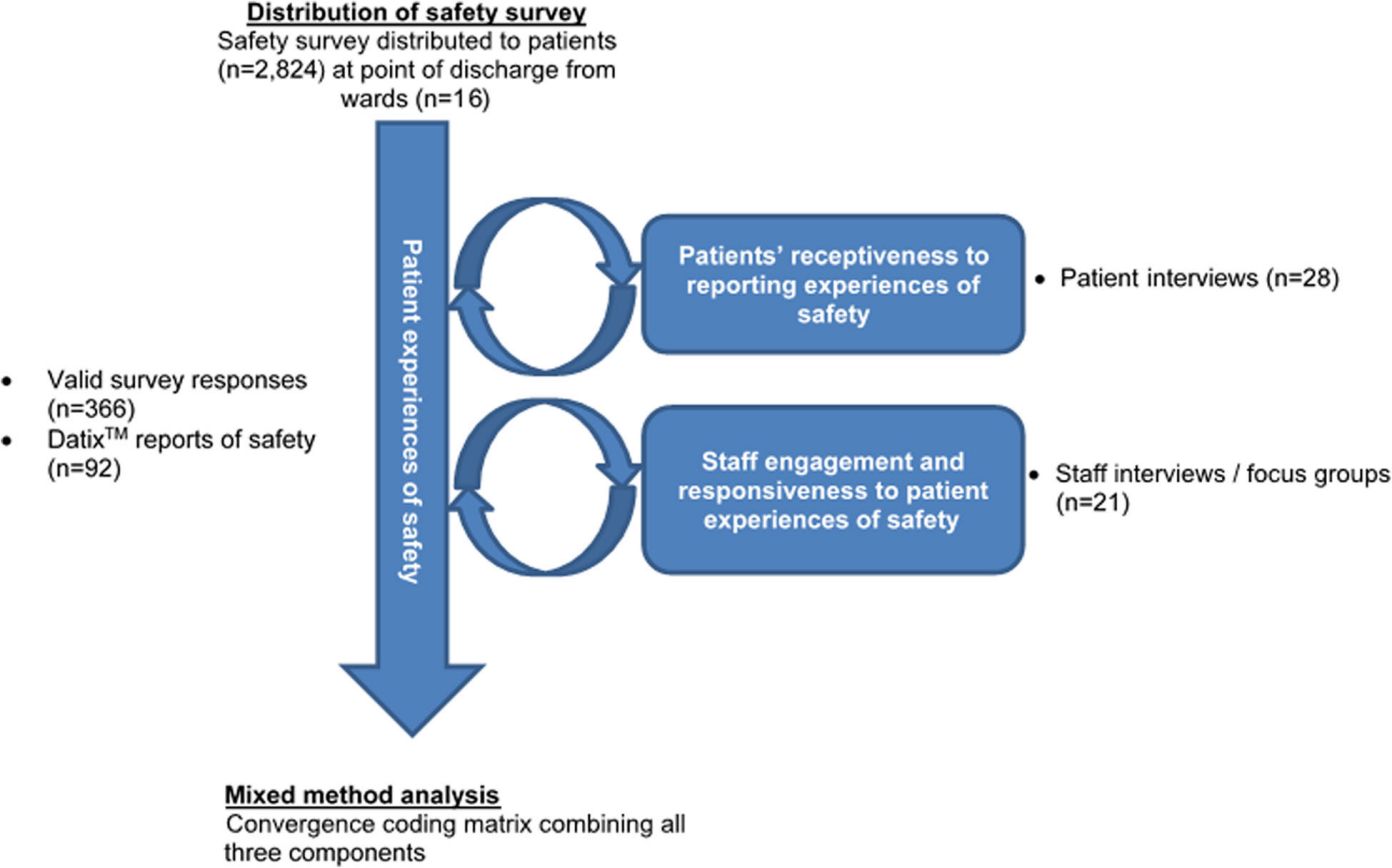
Data collection overview

The study utilised a mixed-methods approach, with quantitative (surveys, distribution rates, response rates) and qualitative (semi-structured interviews, focus groups, staff incident reports) data collected. Distribution of the survey was split into two distinct cycles consisting of 6 months of data collection each. Cycle 1 was conducted from March 2014 to August 2014 and cycle 2 was conducted from January 2015 to June 2015. Data collection was split into the two cycles to allow for changes to be made to the survey as a result of patient feedback (Fig. 1). Information regarding membership of the survey co-design team and the processes of development and validation of the survey have been published elsewhere, including how the survey was amended between cycles 1 and 2 [35].

### Setting

The study was conducted in four hospitals (two general hospitals and two teaching hospitals) from two National Health Service (NHS) Trusts in England. Four clinical areas were chosen in collaboration with the NHS Trusts as the wards that best represented the older population with whom the survey was initially developed [16, 35], and as older patients are at increased risk of safety incidents [36] and are recognised as high priorities in healthcare policy [9]. The four clinical areas, cardiac, care of older people, orthopaedics, and stroke, were represented across 16 wards. Access to the wards was negotiated by site facilitators on behalf of the research team, who discussed the research with ward sisters approximately 3 months before distribution of the survey began.

### Description of safety survey

Both iterations of the safety survey (Additional file 1, or upon reasonable request from the corresponding author) were co-designed by healthcare professionals and expert patients from within the target population of older people, as reported elsewhere [19, 30]. Both versions provided a brief explanation of patient safety and captured patient reports of safety experiences across three stages of the care transfer (*discharge*, *journey* and *arrival or admission*). The questions in surveys distributed in both cycles (described in the study design) focused on six domains of safety; *communication*, *responsiveness*, *waiting times*, *falls*, *medication* and *hygiene*. Patients or their carers were asked to report three levels of safety; *safe* (green), *neutral* (yellow) and *unsafe* (red), and to leave any non-applicable sections blank. Space for free-text comments was provided in both iterations. In the version of the survey distributed in cycle 1 this came in the form of questions asking if there was another reason they felt safe or unsafe, and if anything could have been done to make the patient feel safer. In the version distributed in cycle 2 there was space provided alongside each domain of safety for respondents to expand upon their answers in relation to that specific domain.

The safety survey was provided to patients at the point of discharge, by a member of the clinical team or an administrator responsible for compiling discharge information, e.g. discharge coordinator or ward clerk. Responsibility for distributing the survey was discussed and agreed with the ward sister prior to the start of the study. Patients were provided a letter of invitation to the research study, the safety survey and an evaluation form (Table 1) within a pre-paid envelope, addressed to be returned to a named person from the research team. Pre-paid addressed envelopes were used as they have been shown to improve response rates to surveys [37]. Those distributing the safety survey were asked to prompt the patient to complete and return the safety survey upon arrival at their next location.

**Table 1:** Evaluation form items and response modes

Item number	Item	Response mode
1	I understood the purpose of the Safety Survey	Likert scale, 1–5 (1 = Agree, 3 = Neither Agree or Disagree, 5 = Disagree)
2	I understood what was meant by ‘your recent transfer’	Likert scale, 1–5 (1 = Agree, 3 = Neither Agree or Disagree, 5 = Disagree)
3	I understood each of the questions	Likert scale, 1–5 (1 = Agree, 3 = Neither Agree or Disagree, 5 = Disagree)
4	The questions asked accurately captured what made me feel safe or unsafe	Likert scale, 1–5 (1 = Agree, 3 = Neither Agree or Disagree, 5 = Disagree)
5	There was nothing missing from the Safety Survey	Likert scale, 1–5 (1 = Agree, 3 = Neither Agree or Disagree, 5 = Disagree)
6	I did not experience difficulties completing the Safety Survey	Likert scale, 1–5 (1 = Agree, 3 = Neither Agree or Disagree, 5 = Disagree)
7	I felt that the colour scheme was useful	Likert scale, 1–5 (1 = Agree, 3 = Neither Agree or Disagree, 5 = Disagree)
8	The size of the text was appropriate	Likert scale, 1–5 (1 = Agree, 3 = Neither Agree or Disagree, 5 = Disagree)
9	The Safety Survey allows me to provide useful feedback about the healthcare I have received	Likert scale, 1–5 (1 = Agree, 3 = Neither Agree or Disagree, 5 = Disagree)
10	By receiving this form I feel I am more educated about patient safety	Likert scale, 1–5 (1 = Agree, 3 = Neither Agree or Disagree, 5 = Disagree)
11	Please use the space to expand on your answers or say anything about the survey that you think is relevant	Free-text

Participants opted-in to the study upon completion and return of the safety survey and/or an evaluation form. The option to return either was designed to reduce bias from those who perceived the safety survey negatively and did not wish to complete it, i.e. patients could complete the evaluation survey and opt-in to interviews without returning the safety survey. In the invitation letter and survey, patients’ family members or carers were also encouraged to assist the patient to complete the survey where appropriate, or to complete it on their behalf. Return envelopes contained a unique identifying number to track the ward from which the patient was discharged, and the month of discharge.

### Quantitative data

#### Patient reports of safety experiences (surveys)

Responses to the safety survey were recorded at ward and clinical area levels. Descriptive statistics were compiled for each cycle, the domains of safety, and the stage of the transfer. Non-parametric Kruskal-Wallis and Mann Whitney U tests were used to test for differences in safety ratings based on the clinical areas, and Spearman’s rho correlations were used to determine correlations between age and gender of respondents, and safety ratings.

#### Safety survey distribution rates

At the end of each month, unused surveys were collected. Distribution rates were then calculated as the proportion of all discharges (excluding deaths and in-hospital transfers) given a survey during each month of distribution and are reported descriptively. Discrepancies in distribution figures that resulted in distribution figures of > 100% were identified for two wards. They were excluded from the analysis of distribution rates as this was deemed to be the result of the research process (the use of numbered envelopes to monitor distribution), and not relating to feasibility (acceptability of the survey to staff).

#### Safety survey response rates

A response rate was calculated based on the proportion of surveys returned (numerator) to the number of surveys distributed (denominator). Survey respondents’ demographics (age and gender) and demographic data from wards were combined into weighted clinical area-level data. Wards with only one respondent were excluded from the weighting calculations. For age, data included minimum, maximum, mean and standard deviation. As with distribution rates, two wards were removed from the response rate calculation due to data discrepancies.

### Qualitative data

#### Patient interviews

Semi-structured interviews were conducted by EH (PhD, Research Associate) with 28 patients who completed the safety survey and/or evaluation form. Participants were informed of the reason for the study including the researcher’s role on the project, and provided informed consent. Interview questions included a focus on barriers and enablers to providing useful feedback on their own safety within care transfers and also included general health questions, general safety questions and questions relating to their experience of care transfers. Participants were not asked to comment on or review transcripts. The analysis of these interviews has been published previously in relation to the barriers and facilitators to patients providing feedback [34]. As such, only reflections on the implications of this data for feasibility will be discussed in the results.

#### Staff interviews

Semi-structured interviews using a topic guide (Additional files 2 and 3), conducted in the participant’s place of work or via telephone, and a focus group were conducted by EH (PhD, Research Associate), JS (PhD, Chief Investigator) and ADB (PhD, Research Associate) with 21 staff members who were involved in the transfer of patients or who received the patient feedback. Interview length ranged from 14 min 17 s to 50 min 24 s (mean 28 min 5 s) and focus group length was 56 min 18 s. Participants were informed of the reason for the study including the interviewer’s / facilitator’s role on the project, and provided informed consent. Participants were not asked to comment or review transcripts. The inclusion criteria for staff were that they:

- Work on one of the included wards during the period where safety surveys were distributed, where:
  - ◦ They were responsible for managing the ward, or;
  - ◦ They had been involved in distributing the safety survey, or;
  - ◦ They had responsibility for discharging patients
- Had responsibility for the management of patients or services relating to the transfer of the patient

Questions were structured into three themes; general questions (*job role/title, team, time spent in role/qualified*), general patient safety questions (*understanding of patient safety, role of patients in patient safety, and role of patients in providing feedback on their safety*) and questions about safety survey feedback (*contact with feedback; how feedback had been used in practice (for the ward-based staff), including the relevance and appropriateness of information provided; and the barriers or enablers to using the feedback to learn about patients’ perceptions of safety and improve services*). Where data was collected post-survey distribution or in community care teams, a vignette based on patient feedback was developed to facilitate these discussions. Data collection stopped when it was felt data saturation had been reached.

Interviews were recorded and transcribed verbatim, then coded and analysed systematically using qualitative analysis software. Quotations are reported verbatim and only corrected for spelling and grammar where the meaning is not ambiguous. Staff data were thematically analysed using a deductive and iterative approach by one researcher (ADB), with themes and codes independently verified by the rest of the research team. Drawing on the approaches outlined by Braun and Clarke [38], all transcripts were closely read and initial codes generated and recorded using NVivo software. After initial coding, codes were refined and combined into overarching themes. The themes were refined and finally arranged into larger conceptual groupings. The final codes and themes were verified by all authors. Participants were not invited to provide feedback on the final themes.

#### Staff incident reports

Staff safety incident reports relating to discharge were identified from the Trusts’ Datix incident reporting system for the sixteen wards participating in survey distribution. This included reports that had been assigned ‘failure/delay of discharge’ and ‘admission/transfer problems’. A keyword search developed in conjunction with the patient safety teams was also used to identify incident reports relating to discharge but not included in the pre-existing categories. The keywords were ‘discharge’, ‘transfer’, ‘handover’ and ‘hand-off’. Staff incident reports were provided to the research team in a spreadsheet that contained an incident number, the incident report, action taken, date of incident, category, severity and ward name. Identifiable patient information was removed by the Trusts prior to sharing with the research team. Analysis consisted of JS thematically coding the content of the incident reports and actions taken. The original themes were then grouped into meta-themes and revised to remove any duplication. The final meta-themes and themes were discussed with and approved by JW and PD.

#### Mixed methods analysis

To incorporate the qualitative and quantitative data into a single analysis to provide a triangulated account of the results, key outcomes from all individual analyses were compiled into a convergence coding matrix, which displays results from each component on the same page [39]. For both the qualitative and quantitative data, the results were entered into the matrix as a brief summary by JS. The matrix allowed for an analysis of (dis) agreements, partial (dis) agreements or silences across the different components of the study, which were discussed and populated by JS and ADB before wider discussion amongst all authors.

## Results

The results are presented in relation to the three areas of focus of the feasibility testing: *limited-efficacy testing*, *acceptability*, and *integration*.

### Limited-efficacy testing

#### Patient reports of safety experiences via the survey

A total of 366 patients completed and returned a valid safety survey, defined as one or more complete questions. Analysis of all questions revealed similar patterns amongst all three stages of the transfer (discharge, Table 2; journey, Table 3; arrival, Table 4), suggesting that patients did not differentiate between the stages. Delays were often the largest safety concern for patients, which was reflected in accompanying free-text comments which, where provided, contextualised the ratings provided by the patients.

**Table 2:** Safety survey responses in relation to the departure stage of the transition

Departure		Safety rating			Differences in Characteristics		
	N (% of all 366 respondents)	Safe (%)	Neutral (%)	Unsafe (%)	Clinical area^a^	Age^b^	Gender^b^
Communication	346 (94.5)	304 (87.9)	32 (9.2)	10 (2.9)	*p* = 0.808	*p* = 0.132	*p* = 0.607
Responsiveness	342 (93.4)	303 (88.6)	31 (9.1)	8 (2.3)	*p* = 0.075	*p* = 0.285	*p* = 0.807
Delays^c^	257 (70.2)	Cycle 1: 118 (64.8) Cycle 2: 34 (45.3)	Cycle 1: 51 (28) Cycle 2: 23 (30.7)	Cycle 1: 13 (7.1) Cycle 2: 18 (24.0)	Cycle 1: *p* = 0.874 Cycle 2: *p* = 0.151	*p* = 0.097	*p* = 0.768
Falls	310 (84.7)	268 (86.5)	37 (11.9)	5 (1.6)	p = 0.874	*p* = 0.887	*p* = 0.184
Medication	335 (91.5)	278 (83.0)	36 (10.7)	21 (6.3)	*p* = 0.107	*p* = 0.650	*p* = 0.182
Hygiene	351 (96.0)	319 (90.9)	29 (8.3)	3 (0.9)	*p* = 0.841	*p* = 0.559	*p* = 0.322

^a^ Kruskal-Wallis test comparing the four clinical areas: cardiac, care of older people, orthopaedics, stroke

^b^ Spearman’s rho correlation with safety rating

^c^ Reported per cycle due to changes in the question

**Table 3:** Safety survey responses in relation to the journey stage of the transition

Journey		Safety rating			Differences in Characteristics		
	N (% of all 366 respondents)	Safe (%)	Neutral (%)	Unsafe (%)	Transport type^a^	Age^b^	Gender^b^
Communication	231 (63.1)	213 (92.2)	14 (6.1)	4 (1.7)	*p* < 0.001 Safe Ambulance, 93.3% Private car, 91.0% Patient transport, 85.7%	*p* = 0.121	*p* = 0.876
Responsiveness	230 (62.8)	207 (90.0)	20 (8.7)	3 (1.3)	p < 0.001 Safe Ambulance, 90.8% Private car, 83.3% Patient transport, 66.7%	*p* = 0.911	*p* = 0.463
Delays	226 (61.7)	Cycle 1: 151 (73.5) Cycle 2: 34 (45.3)	Cycle 1: 29 (19.2) Cycle 2: 23 (30.7)	Cycle 1: 11 (7.3) Cycle 2: 18 (24.0)	p < 0.001 Safe^c^ Ambulance, 71.4% Private car, 67.2% Patient transport, 58.3%	*p* = 0.460	*p* = 0.038 (male more likely to report safe)
Falls	230 (62.8)	194 (84.3)	29 (12.6)	7 (3.0)	*p* = 0.009 Safe Ambulance, 90.8% Private car, 83.3% Patient transport, 66.7%	*p* = 0.420	*p* = 0.501
Medication	226 (61.7)	197 (87.2)	23 (10.2)	6 (2.7)	*p* = 0.001 Safe Ambulance, 87.7% Private car, 87.2% Patient transport, 91.7%	*p* = 0.194	*p* = 0.444
Hygiene	232 (63.4)	211 (90.9)	18 (7.8)	3 (1.3)	*p* < 0.001 Safe Ambulance, 91.7% Private car, 92.4% Patient transport, 81.8%	*p* = 0.536	*p* = 0.703

^a^ Kruskal-Wallis test comparing the three categories with > 10 responses: ambulance, private car, patient transport

^b^ Spearman’s rho correlation

^c^ Cycles 1 and 2 combined

**Table 4:** Safety survey responses in relation to the arrival stage of the transition

Arrival		Safety rating			Differences in Characteristics		
	N (% of all 366 respondents)	Safe (%)	Neutral (%)	Unsafe (%)	Arrival destination^a^	Age^b^	Gender^b^
Communication	235 (64.2)	219 (93.2)	11 (4.7)	5 (2.1)	*p* = 0.980	*p* = 0.840	*p* = 0.122
Responsiveness	237 (64.8)	210 (88.6)	23 (9.7)	4 (1.7)	*p* = 0.315	*p* = 0.691	*p* = 0.207
Delays	223 (60.9)	Cycle 1: 118 (79.7) Cycle 2: 34 (45.3)	Cycle 1: 21 (14.2) Cycle 2: 23 (30.7)	Cycle 1: 9 (6.1) Cycle 2: 18 (24.0)	p < 0.001 Safe^c^ Home, 58.8% Hospital, 68.8%	*p* = 0.084	*p* = 0.039 (male more likely to report safe)
Falls	241 (65.8)	204 (84.6)	32 (13.3)	5 (2.1)	*p* = 0.052	*p* = 0.069	p = 0.001 (male more likely to report safe)
Medication	239 (65.3)	213 (89.1)	21 (8.8)	5 (2.1)	*p* = 0.433	*p* = 0.404	*p* = 0.400
Hygiene	241 (65.8)	219 (90.9)	17 (7.1)	5 (2.1)	*p* = 0.779	*p* = 0.927	*p* = 0.351

^a^ Mann-Whitney U test comparing the two categories with > 10 responses: home, hospital

^b^ Spearman’s rho correlation

^c^ Cycle 1 only as too few respondents (n = 2) reported going to hospital in cycle 2

There were no significant correlations between safety ratings and age of respondents across any domain or stage of the transfer. Gender was significantly correlated with safety in relation to delays during journey and arrival, and in relation to falls during arrival, with men more likely to feel safe. Notably, this correlation was non-significant during discharge. The clinical area of discharge also showed no significant correlation with safety ratings. Transport type was correlated with safety ratings; patient transport service (rather than ambulance or private car) was frequently associated with lower perceptions of safety in relation to all six safety domains. Statistics are reported in Tables 2, 3 and 4.

#### Staff incident reports

Three hundred seventy-five staff incident reports submitted during the study period were identified. Following screening by JS, 92 (24.5%) incidents were deemed eligible for inclusion; the remainder of reports examined did not pertain to the patient’s discharge. Thematic analysis of the incident description resulted in eight themes being derived from the data; *communication failures*, *delayed discharge*, *documentation*, *medication*, *pressure ulcers*, *devices or equipment*, *staffing shortages* and *patient actions*. Table 5 presents the staff incident report themes. Of the eight themes, five were novel, in that they were not presented in the safety survey, nor mentioned by any patient participants in the free text sections (*documentation, pressure ulcers, devices or equipment, staffing shortages, and patient actions*).

**Table 5:** Themes and sub-themes of staff incident reports (*n* = 92) relating to patient discharges

Major theme	Sub-theme
Communication failures	• Care home not informed of discharge • Difficulty booking transport • Discharge letter contained incorrect information • Handover not completed properly • Referral to other services not made • Discharged without test results
Delayed discharge	• Result of communication error during booking of transport • Family cause of a delay • Internal delays to medication • Patient transport service aborted or late
Documentation	• Missing documentation • Incomplete documentation • Mistake in documentation • Received wrong patient’s documentation (data breach)
Medication	• Inappropriate medication • Incomplete medication • Incorrect dosage / prescription / dispensation • Missing or lost medication • Patient received someone else’s medication
Pressure ulcers	• Identified prior to discharge • Identified after discharge
Devices / equipment	• Device left in situ after discharge • Incorrect equipment given to patient
Staffing shortages	*No sub-theme*
Patient actions	• Verbal/physical aggression or harassment • Self-discharge against advice • Patient refused discharge

#### Using feedback for organisational learning

Staff who participated in interviews or focus groups (*n* = 21; see Table 6 for participant characteristics) felt that the specific feedback from this survey could be used for learning on both an individual and organisational level, though no evidence of organisational learning was identified during the study.

**Table 6:** Staff participant characteristics

Participant	Participated during or post- survey distribution	Data collection method	Demographics		
			*Gender*	*Clinical area / Speciality*	*Role*
1	During	Interview	Female	Orthopaedic	Senior Ward sister
2	During	Interview	Female	Stroke	Discharge co-ordinator
3	During	Interview	Female	Cardiology	Ward sister
4	During	Interview	Female	Stroke	Discharge co-ordinator
5	During	Interview	Female	Cardiology	Ward administrator
6	During	Interview	Female	Orthopaedic	Ward sister
7	During	Focus group	Male	Stroke	Ward receptionist
8	During	Focus group	Female	Orthopaedic	Apprentice
9	During	Focus group	Female	Orthopaedic	Nurse (band 5)
10	During	Focus group	Female	Orthopaedic	Deputy Sister
11	Post	Interview	Female	Care of Older People	Ward manger
12	Post	Interview	Male	Site facilitator	Patient safety lead
13	Post	Interview	Male	Site facilitator	Senior Research Nurse
14	Post	Interview	Female	Care of Older People	Ward Sister
15	Post	Interview	Male	Site facilitator	Senior Research Nurse
16	Post	Interview	Female	Ambulance service	Patient relations co-ordinator
17	Post	Interview	Female	Care of Older People	Nurse (band 6)
18	Post	Interview	Female	Cardiology	Discharge co-ordinator
19	Post	Interview	Female	Cardiology	Ward sister
20	Post	Interview	Female	Community Care	Occupational Therapist
21	Post	Interview	Female	Community Care	Community Matron

Recognising that most of the safety domains were reported as safe by patients, staff described themselves encouraged by the feedback and found it to be a useful indicator of patient perceptions of safety. The feedback data was also perceived as having the potential to provide a valuable insight into the impact of discharge processes of which staff would otherwise be unaware.

*“I think it would be nice to see ‘cos if a patient has had a good experience on the ward … it would be nice to know that it has carried on afterwards. Cos as I say we try to put everything in place for when they get home or where they’re going, so it would be nice to know that that has carried on, actually worked.”* (Participant 2)

Furthermore, one individual reflected that feedback contained information that addressed issues that had not been considered from a safety perspective, in particular by taking a proactive approach to safety by involving the patient in a meaningful discussion.

*“Just because I know that something is safe, doesn’t necessarily mean that it feels safe to my patients. If it doesn’t feel safe, then, to a degree, I’ve failed … . Even if something isn’t actually unsafe, the interpretation of it is just as important. It has to feel safe, it has to feel like a safe environment.”* (Participant 17)

Survey feedback, specifically where it was positive, was recognised as an important opportunity to commend staff for positive patient experiences of safety and as a tool to bolster and reinforce current good practice. This was especially so as a persistent sentiment existed amongst staff that the wider health system tended to focus attention on negative events and patient safety incidents, rather than also acknowledging what works well. This negative focus, or deficit model of patient safety akin to Safety-I [40], was described as a limited and limiting perspective when there was often scope for sharing best practice among staff. Consequently, this emphasis on mistakes and errors was said to impact considerably on staff morale.

*“It was encouraging to see that actually most people, most of the time - you’re hearing responses that are quite positive, and that’s a good thing.”* (Participant 15)

*“Some of those things [that could be useful] are ones that I wouldn’t have thought to ask someone how safe do they feel about the possibility of falling. That’s probably not something that I would think to ask a patient who was going, to be honest.”* (Participant 11)

*“I think the problem is NHS, really isn't always interested in things that go well. Not to be too negative, but people don’t ever focus on the things that go well. People only ever seem to be focused on things that haven’t gone well, and they’re the things that you hear about and read about more.”* (Participant 11)

Many participants commented that the results of the survey broadly reflected their expectations regarding the issues that created most problems or concerns amongst patients. Overwhelmingly, it was agreed that delays are the main issue for patients and participants felt this result was representative of their experience in the discharge process. Whilst some reported they were basing this assumption on anecdotal evidence, some sites were conducting research to provide insight into this and confirmed that the survey results closely aligned with their investigations. This signifies that patients provided useful and valid feedback that, as a minimum, provides confirmation of anecdotal evidence.

*“There are no big surprises there for me, to be quite honest. I would imagine that the delays section is the biggest issue for everybody going home. That’s not a surprise to me. Loads of people, just anecdotally, complain about how long it takes to get the drugs up and all that sort of thing.”* (Participant 13)

However, several participants also stated there was limited value to only having quantitative data in understanding important safety issues. It was expressed that, while the results were informative in highlighting potential issues as well as areas of excellence, qualitative feedback in the form of patient narratives and quotes was often more effective in resonating with staff and developing a better understanding of the safety concern, issue or incident. This deeper understanding was considered a crucial step in understanding the problem before changes could be suggested or made.

*“Yes, I think [quantitative survey data] adds an important dimension, but probably needs to be not looked at in isolation … What it does is show that these are areas that we should perhaps dig into more. I don’t think it gives you enough information to understand what the real issues are in order to then say, ‘Right, well, we need to look at making these improvements.’”* (Participant 12)

### Acceptability

#### Patient acceptability of providing safety experience feedback

The patient interview data, specifically relating to barriers and facilitators to providing feedback on safety experiences, has been reported elsewhere [34]. To summarise, providing safety experience feedback was acceptable to patients, subject to certain conditions being met. These conditions are represented by three themes, which are combined into a staged model; *cognitive-cultural*, *structural-procedural*, and *learning & change*. The first theme, *cognitive-cultural*, captured the notion that for safety feedback to be deemed acceptable, patients had to understand and prioritise patient safety. The second theme, *structural-procedural*, signified the need for patients to be provided with the opportunity, means and ease of providing feedback, without fear of reprisals, while the individual patient needed the ability and inclination to do so. The third theme, *learning & change*, represented the closure of a feedback loop with patients; they had to feel that their feedback would be acted upon and make a difference to patient safety.

#### Patient acceptability as represented by survey response rates

Estimation of response rates suggest a minimum response rate of 16.4%. Three clinical areas had similar response rates (cardiology, 20.4%; orthopaedics, 22.4%; and stroke, 17.4%), whereas the care of older people clinical area had a much lower response rate of 4.6%. Due to the method of recording distributions these are likely to be an underestimate. This is due to identifiable discrepancies in distribution figures (explained previously) for two wards, where the total number of surveys apparently distributed exceeded the number of discharges.

From the valid surveys returned, 296 (80.9%) surveys were completed by the patient, ten (2.7%) were completed by a carer and two (0.5%) were completed by both patient and carer. The remaining 58 (15.8%) did not state who had completed the survey. 133 participants were female and 160 were male. Participants’ mean age was 64.9 (range = 19 to 96, SD = 15.4). Gender and age of respondents were largely representative of the clinical areas from which they were discharged (Table 7). The exceptions were care of older people (respondents more likely to be younger and female) and stroke (respondents more likely to be younger). Together with the lower response rates from the care of older people clinical area, this is suggestive that older age was a contributing factor to lower responses.

**Table 7:** Comparison of demographics (age, gender) between survey respondents and all patients discharged

	Age					Gender		
	*Survey respondents*			*All discharges*		*Survey respondents*		*All discharges*
Clinical Area (total number of discharges)	*Eligible*	*Weighted mean age, years (std dev)*	*Age Range*	*Weighted mean age, years (std dev)*	*Age Range*	*Eligible*	*Weighted gender*	*All discharges weighted gender*
Cardiology (3318)	145	66.8 (12.4)	28 to 96	66.2 (15.0)	19 to 100	138	50% male 50% female	54% male 46% female
Care of Older People (2947)	16	77.4 (5.7)	68 to 93	84.6 (6.1)	41 to 105	17	31.2% male 68.8% female	52.7% male 47.3% female
Orthopaedics (3859)	108	60.1 (15.0)	19 to 88	62.8 (17.5)	16 to 105	115	66.1% male 33.9% female	53.6% male 46.4% female
Stroke (1260)	22	62.1 (20.6)	21 to 91	74.3 (13.9)	21 to 103	21	45% male 55% female	43.8% male 56.2% female

#### Staff acceptability of patients providing safety experience feedback

Analysis of interview data showed that staff valued patient feedback on their safety experiences as serving to improve staff awareness of safety as well as acting as an additional barrier in the prevention and minimisation of harm.

*“I think, yes, obviously the more we know about things like [the patient’s experience of safety], the more we can do to reduce the risks of patients being injured or something happening with patient safety relating to our care, I think yes, it [their feedback] would be valuable”.* (Participant 16)

Spending time and communicating with patients was perceived to encourage patients to provide feedback on their safety experiences. The quotation by Participant 15 demonstrates that resources are important to making meaningful connections with patients.

*“Of course that’s the big C word, communication. It’s all about making sure people have got the information in a format they can understand. That we’re not patronising, not making assumptions about what people know and don’t know. You have put up frank explanations for things, and we check out what people have understood.”* (Participant 15)

There was also a persistent belief among interviewees that older adults were generally less likely to report any issues or concerns and were more likely to trust the care team without question. One individual stated that some members of the older generation were *“inappropriately trusting of the system”* (Participant 15) and reluctant to be perceived as causing a *“fuss”* (Participant 16) or to question the clinicians’ decisions. There was also concern expressed that older adults were less likely to complain due to *“the thought of having to take on a bigger organisation”* (Participant 16).

*“[Older people] never really want to say anything negative, but I think that’s just because of the age that they are. It’s that generation.”* (Participant 11)

### Integration

#### Integration of the survey with existing systems and practices

Staff discussed their role in facilitating the collection of patient feedback on safety, identifying numerous facilitators and barriers to doing so. Staff prompting and reminding each other was deemed helpful to facilitate distribution and maintain and boost distribution rates, as was making the survey visible and easily accessible. Thus, those sites in which survey distribution was considered a team endeavour, with staff working together to remind and encourage each other to distribute the survey, appeared to be most successful in distribution.

*“I think it’s just trying to prompt each other sometimes... I mean it depends who's on ‘cause everybody's different really, but I mean what I like to do is try and sort of prompt, you know like, ‘Ooh you could’, you know, ‘have given them that as well’ and you kind of get people who'll remind you.”* (Participant 5)

Barriers to integration included resource limitations (especially nurses’ own time) and staff turnover.

*“I think it’s a bit unfair to ask the nurses to do anymore, personally, do you know what I mean? But not everybody has a discharge co-ordinator and I think probably in the absence of the discharge co-ordinator there’s probably the receptionist that could do it, but I think nursing staff I just think sometimes they’ve got too much on the plate to ask”* (Participant 18)

Another barrier to integration was a reluctance amongst staff to overburden patients with paperwork, particularly during discharge when they are deemed to be vulnerable.

*“I feel they get bombarded sometimes with information and things that they need to do and all they want to do is just get home, and once they’re home, I don’t know, they might not want to complete them, complete any surveys. I mean I’m sure if they thought it was going to help patients in the future then they might think differently about it, but I know just from feedback we’ve had about surveys, they do find it a bit much completing lots of paperwork.”* (Participant 6)

#### Integration as represented by survey distribution rates

Eleven thousand two hundred eighty-two patients were discharged from the included wards. It was not possible to determine the exact distribution rate as some surveys that had not been distributed may not have been returned to the research team because, for example, they had been lost or thrown away on the ward. As such, there were a maximum of 2824 (25.1%) surveys distributed, though the actual number was likely lower. Distribution rates varied amongst clinical areas (cardiology, 30.5%; care of older people, 28.3%; orthopaedics, 19.7%; stroke, 20.0%). There was also large variation in distribution rates at ward level (9.2 to 46.3%) regardless of the clinical area, suggesting that variables other than the clinical area or the NHS Trust were responsible for variation.

## Discussion

The aim of this study was to determine the feasibility of implementing a patient safety survey which measures patient experiences of their own safety relating to care transfer. In particular, the study explored *limited-efficacy testing, acceptability* (to patients and staff) of the safety survey, and *integration* with existing systems and practices from the staff perspective.

From the limited-efficacy testing, patient reports on their experiences of safety identified that delays relating to departure from hospital made patients feel least safe. Where patients identified the cause of this feeling, it was often associated with delays in obtaining medication or from the lack of explanation and reassurance from staff about the delay. However, there were discrepancies between patient reports and staff reports, with patients identifying aspects of their care that made them unsafe which staff did not report, and staff identifying types of incidents that patients did not report, including incidents caused by patients. These results reflect existing evidence that patients and staff are able to identify some of the same safety issues, but also identify different safety issues [29–31]. This study expands on the existing literature by demonstrating this within the organisational care transfer setting, thus providing support for the notion that patients can provide constructive feedback on their experiences and perceptions of safety in this context. These results also demonstrate that it is possible to collect meaningful data relating to safety experiences from patients in relation to their care transitions.

Qualitative data from staff demonstrated a degree of staff acceptability to using the survey, including belief that patient feedback from the survey could be used for service improvement, which in turn can contribute to a culture of continuous learning. Quantitative feedback was seen to serve the purpose of indicating where there may be problems, and qualitative feedback to inform the types of changes required to improve care. However, staff within this study did not appear to engage in quality improvement activities based on patient experiences of safety, and we are therefore only able to conclude that patient feedback on their safety could lead to quality improvement, but that other individual, structural, procedural and cultural conditions are required to be met first. This supports existing research that patients should be involved in the improvement process, providing their involvement is managed correctly [41, 42] and they welcome having some responsibility for their safety [43]. For instance in one study [44] using a national patient survey for quality improvement, it was identified that staff were largely receptive to the survey results but that there were a number of barriers. These barriers included survey results that were not directly meaningful to individual wards or units, and limited resources or knowledge to make changes [44]. Evidence also suggests that providing written feedback to wards is not sufficient for enabling quality improvement, even if that feedback is specific to the ward [45]. We identified a similar barrier in the context of distribution of feedback tools, in particular the time constraints that impacted upon the distribution of the survey to patients. An additional barrier was the perception that patients would be overburdened with paperwork, thus limiting the opportunity for patients to provide feedback on their safety experiences; this formed a structural-procedural barrier to patients providing feedback [34].

Staff interviews also identified that there was a systematic focus on unsafe or negative experiences of care, which reflects the deficit approach to safety [40] and has been dominant throughout healthcare services since the safety movement began in earnest at the turn of the century [46]. However, some healthcare professionals in the study acknowledged that an appreciative approach to patient safety could help them to understand what it is that they have done correctly. As such, they felt that sharing best practice can lead to quality improvement. However, it was noted that any approach that relied on staff distributing surveys and obtaining and collating feedback, carried a real risk of overburdening those staff, which in turn would hinder any quality improvement efforts.

The results are moderated by conceptual and pragmatic issues relating to the implementation of the survey, which would need to be addressed before implementation - using the approaches taken in this study - could be possible. Further testing to determine whether feedback can contribute to a change in care is required. For instance, patients were able to highlight aspects of their care that had made them feel safe or unsafe, but this was often conflated with other aspects of care (i.e. beyond the transfer from hospital) [34], including the transfer *into* hospital or their experiences on the ward. Such conflation was also reflected in patients often reporting the same ratings of safety across the three different stages of the transfer; based on interviews and open text comments, this was not always representative of how they experienced safety within those individual stages. This suggests that a safety experience from one location of care (for example in the hospital setting) is remembered and reflected in the feedback on latter stages, including transfer.

### Study limitations

There is considerable scope for a Type-I error within the statistics, given the number of variables that were tested. There was consistency in the correlation between transport type and safety ratings across all six domains of safety, which suggests this result was not subject to a Type-I error. However, there was no such consistency in relation to the three significant gender-safety rating correlations, and as such these results should be treated with caution.

There were a number of other limitations to the study, although many of these are indicative of the feasibility nature of the study. The first of these limitations was that the number of responses to the survey and the number and varied categorisation of staff incident reports meant that it was not possible to perform statistical analysis to look for correlations or relationships beyond those that appear at a thematic level. This was also reflected in some subgroups with a small number of participants, such as respondents from care of older people wards (*n* = 16).

A further limitation relates to the limited data obtained from existing measures and indicators of quality and safety. Firstly, with the exception of staff incident reports, there were very few if any routinely collected data that are directly relevant to the care transfer, or the discharge from hospital. Data on patient complaints was obtained, however there were too few for any meaningful statistical analysis or for inclusion in the thematic analysis due to identification concerns. The complaints data only contained a single category, meaning any form of thematic analysis similar to the staff incident reports was not possible. Other routinely collected safety data, such as from the Safety Thermometer [47], was deemed to be irrelevant to the discharge process and was therefore not obtained. Readmission and length of stay data was obtained for eight of the 16 wards, but again there was insufficient data for inferential analysis.

As the research team were not involved in collecting the reports, with the exception of producing the surveys and providing them to participating wards, we encountered a number of barriers to conducting the research that were not specifically related to feasibility of implementing a safety survey. These included a lack of awareness amongst staff on the wards, caused in part by high staff turnover, resistance to change or a lack of motivation to engage, confusion between multiple surveys to give to patients and time or resource constraints. Whilst a more resource-intensive approach could have been used, such as having more research staff to facilitate the distribution of the survey or incentivising the distribution, the results provide a more accurate reflection of what would happen were the survey to be introduced into routine practice.

Finally, the use of numerically-identified envelopes allowed envelopes to be tracked from distribution through to response. However, there were some discrepancies in the distribution data as a result of using this process, as described in the methods. This was usually isolated months rather than over a prolonged period of time, and was accounted for to some extent in the analysis. However it is likely that distribution rates and response rates were influenced by these discrepancies. Specifically, distribution rates will have been lower than identified, and response rates will have been higher.

### Implications for research and practice

Patient experience is recognised as a pillar of healthcare quality [32], but there needs to be sufficient resources to support the collection of experience data so that it does not become a burden for front-line teams. However, removing the onus from front-line staff may generate suspicion of the system and staff disconnectedness, as has happened with the Friends and Family Test in the English NHS [48]. Future research needs to examine whether patient feedback in relation to their safety during transitions in care is able to influence practice and drive quality improvement at the local level. Whilst there is some limited evidence that this may be the case in single care settings [24], and staff within this study reported that it should be possible, there is still a requirement to identify how this can be done in practice where multiple boundaries exist. There is also a need to investigate other factors that contribute to patients’ experiences of safety, such as where patients are transitioning to, and whether treatment is still ongoing or complete.

As patients struggled to differentiate between the different stages of their care, it is necessary to question the assumption that patients are better placed than healthcare professionals - who only see parts of the transfer relevant to their role [7, 13] - to identify safety issues that span multiple boundaries and organisations. Future research should aim to identify the unique aspects of the transition that the patient and care provider can identify both individually and jointly, which would need to include developing a greater understanding of how patients perceive boundaries within health and social care. Such research could move towards providing more comprehensive datasets that link multiple types of feedback from patients and healthcare professionals specific to single episodes of care or transitions, thus providing a more holistic perspective. Current policy drivers towards improved health and social care integration [9] may help with the system changes necessary to facilitate these data sets.

## Conclusions

Limited efficacy testing suggests that patients can provide meaningful feedback on their experiences and perceptions of safety in the context of care transitions. Furthermore, providing safety experience feedback was acceptable to some patients, subject to certain conditions being met; *cognitive-cultural* (patient understanding and prioritisation of safety), *structural-procedural* (opportunities, means and ease of providing feedback without fear of reprisals), and *learning & change* (closure of the feedback loop) [34]. Safety experience feedback from patients was also acceptable to staff, with quantitative data serving the purpose of indicating where there may be problems, and qualitative data informing the types of changes required to improve care. However, patient feedback was not integrated into any quality improvement initiatives, suggesting that there are still significant challenges to healthcare teams or organisations utilising patient feedback, particularly in relation to care transitions.

## Additional files

Additional file 1: Safety Survey. This file contains the final version of the safety survey distributed to patients as part of the limited efficacy testing (PDF 423 kb)

Additional file 2: Patient Interview Topic Guide. This file contains the interview topic guide used with patients. (DOCX 79 kb)

Additional file 3: Staff Interview Topic Guide. This file contains the interview topic guide used with staff members. (DOCX 72 kb)

## Data Availability

Participants did not provide consent to share their data beyond the original study team. The study team would be happy to interrogate the data on behalf of others upon reasonable request to the corresponding author. In line with the study’s ethical approval, restrictions apply to the availability of the data, and it will no longer be available for interrogation after 31st December 2021. Study materials required for replication are available as Additional files 1, 2 and 3.

## Abbreviations

NHS: National Health Service
