# Supplementary material for: Implementing a survey for patients to provide safety experience feedback following a care transition: a feasibility study
Source: BMC Health Serv Res. 2019 Aug 30;19:613. doi: 10.1186/s12913-019-4447-9 (PMC6716906; doi:10.1186/s12913-019-4447-9)
Supplement: Supplementary file 1 — Safety Survey. This file contains the final version of the safety survey distributed to patients as part of the limited efficacy testing (PDF 423 kb) [file 12913_2019_4447_MOESM1_ESM.pdf]

# SAFE AND SURE

## Safety Survey

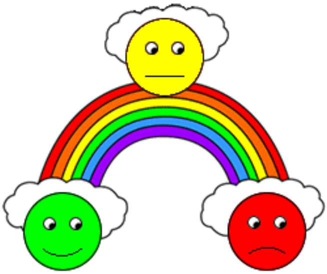

Dear patient and / or carer,

This survey is for you to tell us how safe you felt during your most recent transfer out of hospital, and what made you feel this way. Anything that you tell us will remain confidential and will not affect the care that you receive.

It is important for us to find out about your experiences so that we can improve our services. **Please complete the survey and return it in the prepaid envelope provided**.

Contact Jason Scott or Emily Heavey if you have any questions, would like help completing the survey or if you would like to receive the survey in large print.

**01904 876 376**

j.scott@yorksj.ac.uk

e.heavey@yorksj.ac.uk

**What does safety mean?**
We believe that for you to feel safe, healthcare staff should communicate with you, respond to your individual needs and ensure you are physically safe and secure. We are also interested in finding out if there is anything else that makes you feel safe.

**How do I complete the survey?**
For each question, please tick the face that best represents how you felt. The **green face** means you had no worries or concerns about your safety, the **red face** means you were worried or concerned about your safety, and the **yellow face** means you felt somewhere between the two.

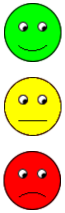

York St John University · York Teaching Hospital NHS Foundation Trust · The University of Nottingham · Nottingham University Hospitals NHS Trust · The Health Foundation Inspiring Improvement · Yorkshire Ambulance Service NHS Trust

**Departure** means planning and preparing for, and leaving hospital.

**Journey** means travelling from hospital to your next location.

**Arrival** means settling in at your next location.

**What is your NHS Number?** (optional) ____________________

**Are these the opinions of:** patient ❑ carer ❑

**What was the date of your departure?** D D / M M / Y Y Y Y

**Which ward did you depart from?** ____________________

**Where were you going to?** ____________________

**How did you get there?** ____________________

**Did someone go with you?** Yes ❑ No ❑

*If yes, who?* Family / Friend ❑ Carer ❑ Member of Staff ❑

Only tick boxes for questions below that are relevant to you, for example the question on staff communication during your journey may not be applicable if you used your own transport.

---

**How safe did the communication from staff make you feel?** For example giving you clear and timely information or being polite.

| | | | | Comments: |
|---|---|---|---|---|
| On your departure | ❑ | ❑ | ❑ | |
| During your journey | ❑ | ❑ | ❑ | |
| On arrival at your next location | ❑ | ❑ | ❑ | |

---

**How safe did you feel with regards to staff listening to you and responding to your individual needs?**

| | | | | Comments: |
|---|---|---|---|---|
| On your departure | ❑ | ❑ | ❑ | |
| During your journey | ❑ | ❑ | ❑ | |
| On arrival at your next location | ❑ | ❑ | ❑ | |

**Did you experience any delays?** Yes ❑ No ❑

**If yes, where was the longest delay during your transfer?**

Departure ❑ Journey ❑ Arrival ❑ Comments: ____________________

**How did this make you feel?**

❑ ❑ ❑

____________________

____________________

**How safe did you feel about the possibility of falling?** For example if you felt confident that you wouldn't fall or if you were you concerned that you might

| | | | | Comments: ____________________ |
|---|---|---|---|---|
| On your departure | ❑ | ❑ | ❑ | ____________________ |
| During your journey | ❑ | ❑ | ❑ | ____________________ |
| On arrival at your next location | ❑ | ❑ | ❑ | ____________________ |

**How safe did you feel about your medication?** For example receiving the correct medication, understanding the medication you were taking or delays in receiving your medication.

| | | | | Comments: ____________________ |
|---|---|---|---|---|
| On your departure | ❑ | ❑ | ❑ | ____________________ |
| During your journey | ❑ | ❑ | ❑ | ____________________ |
| On arrival at your next location | ❑ | ❑ | ❑ | ____________________ |

**How safe did you feel about hygiene and cleanliness?** For example if staff washed their hands and if the surroundings were clean

| | | | | Comments: ____________________ |
|---|---|---|---|---|
| On your departure | ❑ | ❑ | ❑ | ____________________ |
| During your journey | ❑ | ❑ | ❑ | ____________________ |
| On arrival at your next location | ❑ | ❑ | ❑ | ____________________ |

**Overall, how safe did you feel throughout the whole transfer including the departure, journey and arrival?**

Comments: ____________________

❑ ❑ ❑

____________________

____________________

Thank you for taking the time to complete this survey. Please return it in the freepost envelope provided.

**What will we do with your answers to this survey?**
We will bring together feedback from patients and provide this anonymously to healthcare teams involved in your transfer. The purpose of this is to identify what is being done well, and areas where the quality of care that you receive can be improved.

**What should you do if you want to make a complaint about your care?**
By completing this survey you are **not** making a complaint. If you have felt unsafe at any other point during your care or would like to raise a specific concern please contact the Patient Advice and Liaison Service. If you contact us we can give you information on how to do this.

**Would you like to receive a summary of the research findings?**

Yes ❑ No ❑

Please fill out your details below and we will send you this at the end of the study. All information will remain private and confidential in line with the Data Protection Act (1998), and will not be shared with anyone or used for any other purpose than to provide you feedback.

Name: ______________________________________________

Address: ____________________________________________

____________________________________________

Could you please tell us your gender, age and how you define your racial / ethnic origin. This will tell us if we're reaching a wide sample of people. If you are a carer, please tell us the patient's details. You do not have to complete this part if you do not want to.

Gender: Male ❑ Female ❑

Age: ____________________

Racial / ethnic origin: ____________________
