# Supplementary material for: Implementing a survey for patients to provide safety experience feedback following a care transition: a feasibility study
Source: BMC Health Serv Res. 2019 Aug 30;19:613. doi: 10.1186/s12913-019-4447-9 (PMC6716906; doi:10.1186/s12913-019-4447-9)
Supplement: Supplementary file 2 — Patient Interview Topic Guide. This file contains the interview topic guide used with patients. (DOCX 79 kb) [file 12913_2019_4447_MOESM2_ESM.docx]

**PATIENT INTERVIEW SCHEDULE**

**Briefing**

- The purpose of the interview
  - To get an understanding of what they understand about patient safety
  - To get feedback on the reporting tool
  - To find out if it accurately captures their thoughts on safety
  - To get an understanding of how they think the reports of safety can lead to improvements
- Interview will be recorded unless they ask otherwise
- Why they have been asked take part in the study
- How long the interview will last
  - Approximately 30 to 60 minutes
- Their rights as participants
  - Right to withdraw at any time
  - Ask questions at any time
  - Right to complain
  - Anything that is said today will not affect your healthcare
    - What you tell me today will remain completely confidential and you will remain anonymous.
      - However if you tell me something that suggests yourself or someone else may be at risk of harm, I will have to break confidentiality. Again, doing so will not affect the care that you receive.
- Have they got any questions?
- Signing of the consent form

*To begin with I’d like to ask you a few questions about yourself. Remember, if you don’t want to answer a question please say.*

General Health Questions

1. **How old are you?**
2. **Do you consider yourself to have any disabilities**
   1. **If yes, what are they?**
3. **What would you describe your ethnicity?**
4. **What sort of care, if any, are you receiving at the moment?**
5. **Roughly how often do you go into hospital?**

General Safety Questions

1. **In terms of the care that you receive, what do you understand by safety?**
2. **Have you ever been involved in something to do with your care that may have or did affect your safety?**

Prompt

- What about someone else’s safety?

1. **Have you ever experienced something that made you feel particularly safe?**

Prompt

- Think back to the last time you were discharged from hospital

1. **Do you think that as a patient, you should have a role in your own safety?**

Prompt

- What role should you play and why?
- Who should ultimately be responsible for your safety and why?
- Do you think that you can make a difference to your own safety?
  - If yes, how? What would help this to happen?
  - If no, why not? What are the barriers?

1. **Do you think patients should be providing feedback on their safety?**

Prompt

- Will doing so make any difference? Why / why not?
  - If no, ask what would need to change

1. **Can you think of any reasons why patients would or would not be willing to provide feedback on their safety?**

Care Transfer Questions

*I’d now like to ask you some questions about your recent transfer out of hospital. This includes when you were being discharged, the journey or transport to your next destination and when you arrived there.*

1. **Can you tell me about your recent transfer?**

Prompt

- Where were you discharged from? Where were you transferred to? How did you get there?
- Who was involved in your transfer? (can be staff, family, friends etc)
- Relating to your safety, did anything of note happen?

1. **In the survey, you said […]. What was it that made you choose these answers?**
2. **Ask a question about feeling safe**
3. **Ask a question about feeling unsafe**
4. **Would you say that your experiences would make you more or less likely to report on your safety?**

Safety Survey Questions

1. **Can you tell me what you thought of the safety survey in general?**
2. **Did you feel you understood the point of the safety survey?**

Prompt

- What do you think the survey was trying to find out?
- Why do you think we’d what to find out about this?
- Did you think it allowed you to provide useful feedback?

1. **Did you feel that the survey provided you with any useful information about safety?**
2. **Did you experience any difficulties filling out the survey?**
3. **Did you feel you understood what all of the questions were asking you?**
4. **I’d like to go through the safety survey to see how you interpret(ed) it. Can you tell me what you understood by:**
   1. **‘Your departure’**

Prompt

- Departure from where?
- What sorts of events/places might this involve?
  1. **‘Your journey’**

Prompt

- Journey from where to where?
  1. **‘Your Arrival’**

Prompt

- Arrival where?
- What sorts of events/places might this involve?

1. **In terms of your departure, what do you think we might have meant by the following? Can you give an example?**
   1. Communication from staff
   2. Staff listening to you
   3. Departure running to schedule
   4. Falling or potential falls
   5. Medication problems or concerns
   6. Hygiene
2. **In terms of your journey, what do you think we might have meant by the following? Can you give an example?**
   1. Communication from staff
   2. Staff listening to you
   3. Journey running to schedule
   4. Falling or potential falls
   5. Medication problems or concerns
   6. Hygiene
3. **In terms of your arrival, what do you think we might have meant by the following? Can you give an example?**
   1. Communication from staff
   2. Staff listening to you
   3. Waiting times
   4. Falling or potential falls
   5. Medication problems or concerns
   6. Hygiene
4. **Did you think the questions that it asked reflected what you think about safety?**

Prompt

- Why or why not?

1. **Was there anything that it missed?**
2. **Are there any other ways that you think would be more appropriate to provide feedback on your safety?**

Prompt

- Other formats or questions?

1. **Did you think that completing the survey may or may not affect the care that you receive in the future?**

Prompt

- If no, why not? What are the barriers to this?
- If yes, how? What can make this happen better?

1. **Is there anything else that you’d like to add?**
