# Supplementary material for: Implementing a survey for patients to provide safety experience feedback following a care transition: a feasibility study
Source: BMC Health Serv Res. 2019 Aug 30;19:613. doi: 10.1186/s12913-019-4447-9 (PMC6716906; doi:10.1186/s12913-019-4447-9)
Supplement: Supplementary file 3 — Staff Interview Topic Guide. This file contains the interview topic guide used with staff members. (DOCX 72 kb) [file 12913_2019_4447_MOESM3_ESM.docx]

**HCP INTERVIEW SCHEDULE**

**Briefing**

- The purpose of the interview
  - Talk about safety and patient reporting of safety
  - Understand how patient reports of safety can been used
- Interview will be recorded unless they ask otherwise
- Why they have been asked take part in the study
- How long the interview will last
  - Approximately 30 to 60 minutes
- Their rights as participants
  - Right to withdraw at any time
  - Ask questions at any time
  - Right to complain
  - What you tell me today will remain completely confidential and you will remain anonymous.
    - However if you tell me something that suggests yourself or someone else may be at risk of harm, I will have to break confidentiality. Again, doing so will not affect the care that you receive.
- Have they got any questions?
- Signing of the consent form

*To begin with, I’d like to ask you some questions about yourself.*

General Questions

1. **What’s your role / job?**
2. **Which team do you work in?**
3. **How long have you worked in this role / been qualified?**

General Patient Safety Questions

1. **What do you understand by the term patient safety?**
2. **Do you think that patients should have a role in their own safety?**

Prompt

- What role should this be? Why?
- Who should ultimately be responsible for patients’ safety? Why?
- Do you think that patients can make a difference to their own safety?
  - If yes, how? What would help this to happen?
  - If no, why not? What are the barriers?

1. **Do you think patients should be providing feedback on their safety?**

Prompt

- Will doing so make any difference? Why / why not?
  - If no, ask what would need to change

1. **Are there any reasons a patient wouldn’t provide feedback on their safety?**

Prompt

- Reasons for being unwilling, unable or unready to provide feedback
- What can be done to change this?
  - For example what types of support might be needed or is it a wider issue?

Questions about Safety Survey Distribution

1. **How did you experience distributing the surveys to patients?**

Prompt

- Did you find that the survey was distributed to all discharges?
- Was there anything that prevented you or others from distributing the survey?
- Was there anything that helped increase distribution?
- Did distributing the survey interfere with any of your other tasks?
- Does your ward distribute any other surveys (e.g. Friends and Family)? If so, how did the distribution of this survey compare?

1. **What would improve distribution rates of surveys like this one?**

Prompt

- Do staff need more reminders, or a stronger motivation to distribute/explanation as to why distribution matters?
- Can you think of any ways that distribution of the survey could be embedded into regular practice?

Questions about Safety Survey Feedback

*I’d like to now ask you some questions about the safety survey feedback*

1. **What sort of contact have you had with the feedback?**

Prompt

- Were you responsible for receiving and using the feedback? If not, how were you in receipt of the feedback, and by whom?

1. **Can you tell me what you think about the feedback?**

Prompt

- Was it useful? Do you think it accurately reflects things? Were there other questions we should be asking patients?

1. **Have you learned anything from the feedback?**

Prompt

- Can you give some examples?
- [If appropriate] Do you have anything to support this?

1. **Have you made any changes based on the feedback?**

Prompt

- If yes, what changes have you made? How did the feedback help? What could be done better?
- If no, why not? What would need to be done to be able to make these changes?

1. **Are there more appropriate ways for patients to provide feedback about their discharge?**

Prompt

- If yes, what are they? How would they be better?
- If no, why not?

1. **Is there anything else that you’d like to mention?**
